# Added Complexity!—Mechanistic Aspects of Heterobimetallic Complexes for Application in Homogeneous Catalysis

**DOI:** 10.3390/molecules28104233

**Published:** 2023-05-22

**Authors:** Zeno Fickenscher, Evamarie Hey-Hawkins

**Affiliations:** Institute of Inorganic Chemistry, Universität Leipzig, Johannisallee 29, D-04103 Leipzig, Germany; zf68zake@studserv.uni-leipzig.de

**Keywords:** heterobimetallic complexes, mechanism, electronic interaction, steric scaffolding, tandem catalysis, synergistic catalysis

## Abstract

Inspired by multimetallic assemblies and their role in enzyme catalysis, chemists have developed a plethora of heterobimetallic complexes for application in homogeneous catalysis. Starting with small heterobimetallic complexes with σ-donating and π-accepting ligands, such as N-heterocyclic carbene and carbonyl ligands, more and more complex systems have been developed over the past two decades. These systems can show a significant increase in catalytic activity compared with their monometallic counterparts. This increase can be attributed to new reaction pathways enabled by the presence of a second metal center in the active catalyst. This review focuses on mechanistic aspects of heterobimetallic complexes in homogeneous catalysis. Depending on the type of interaction of the second metal with the substrates, heterobimetallic complexes can be subdivided into four classes. Each of these classes is illustrated with multiple examples, showcasing the versatility of both, the types of interactions possible, and the reactions accessible.

## 1. Introduction: Multimetallic Catalysis: From Enzymes to Complexes

Humanity is currently facing two existential and inherently connected challenges: on the one hand, the ever-increasing demand for energy and, on the other hand, the consequences of anthropogenic greenhouse gas emissions [1]. In particular, the increased intensity of the global climate crisis shows the necessity to move away from energy sources based on the combustion of fixed carbon towards carbon-neutral production of fuels [2]. A particularly desirable goal is the direct reduction of carbon dioxide into an energy carrier [3,4]. Considering that energy demand, specifically the global consumption of energy per hour, is predicted to reach 1.1 × 10^21^ J by 2050, of which only 20% could be derived from fossil fuel resources, the need to gain access to new sources of energy is underpinned even more [5,6].

The reduction of carbon dioxide can be thermodynamically unfavorable, depending on the level of reduction, but is always associated with a high kinetic barrier; thus, the use of a catalyst is quintessential [4,7,8,9]. While there are industrial processes that use catalysts for carbon dioxide reduction, to a certain level of success, both their scale and efficiency pale in comparison to biological carbon dioxide fixation (the transformation of gaseous carbon dioxide into higher energy compounds, such as formic acid, methanol, or glucose) [3,10]. The most notable example is photosynthesis, which is arguably the only catalytic process for carbonaceous fuel production on a global scale [11]. The autotrophic organisms performing the reduction of carbon dioxide have developed multiple different pathways to do so. Currently, six conceptually different autotrophic CO_2_ metabolizing pathways are known; the most important of these is the Calvin cycle found in all plants, algae, and cyanobacteria [10,11]. It accounts for ~90% of the biological carbon fixation worldwide, and the key enzyme ribulose-1,5-bisphosphate carboxylase-oxygenase (Ru-BisCO) is the most abundant protein on earth [12].

**Scheme 1:**
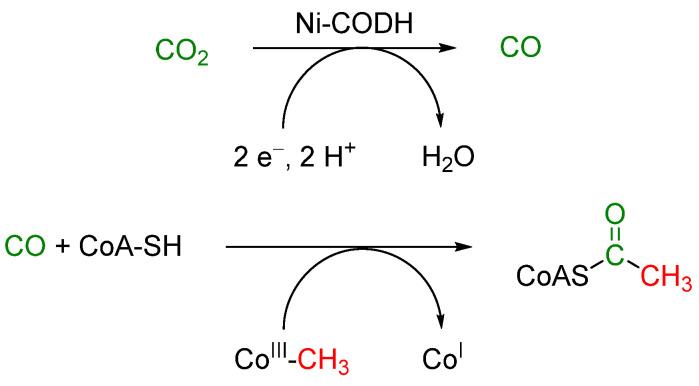
Schematic reduction of CO_2_ to CO during the Wood-Ljungdahl pathway and subsequent incorporation into coenzyme A (CoA-SH), together with Co-containing methyl transferring enzyme (Co^III^-CH_3_) to form acetyl-coenzyme A (acetyl-CoA) [16].

**Scheme 2:**
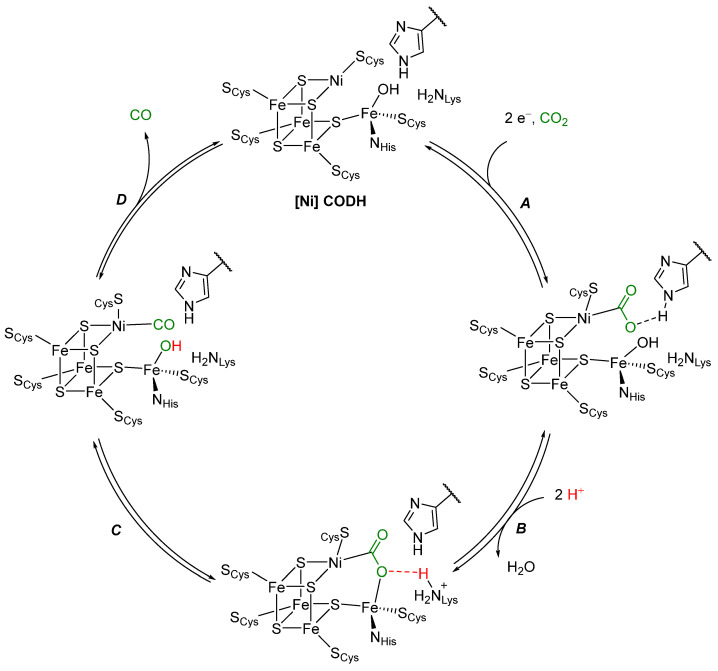
Proposed mechanism of CO_2_ reduction to CO by [Ni] CODH [17,19,20,21].

Originally found in heterotrophic bacteria but now also shown to be significant for chemoautotrophic organisms, such as *methanogens*, the Wood-Ljungdahl pathway is less common [13,14]. However, it is considered to be the oldest of all carbon dioxide fixation pathways and possibly the energy metabolism of the last universal ancestor of all cells [15]. The metabolic product of the Wood-Ljungdahl pathway is acetyl-coenzyme A (acetyl-CoA, Scheme 1), which is produced from coenzyme A, a methyl-transferring enzyme (Co^III^-CH_3_), and carbon monoxide, generated by Ni-dependent carbon monoxide dehydrogenase ([Ni] CODH, Scheme 2) [16].

The active site of [Ni] CODH contains a unique cluster, called the C-cluster. The structure derives from a [4Fe4S] cluster with a nickel replacing one of the iron atoms and an additional iron pendant to the cluster. All the metal atoms in the cluster are ligated by cysteine and a cluster sulfide, while the pendant iron is ligated by a histidine, a cysteine, and a hydroxyl ligand [17]. Four different states of the C-cluster have been spectroscopically identified [18]. Of these four states, two are believed to be the active states for the oxidation of CO to CO_2_. Through kinetic, spectroscopic, and structural studies, the following catalytic cycle for CO_2_ reduction by [Ni] CODH (Scheme 2) is proposed [17,19,20,21]. A two-electron process (***A***) likely occurs via an *ECE* mechanism: First, an *electron transfer* to form Ni^I^ (E), followed by a *chemical step* (C), binding of CO_2_ to Ni, and then a second *electron transfer* step (E). Alternative pathways may be possible, resulting in a CO_2_ adduct stabilized by hydrogen bonding with a protonated histidine residue. One of the oxygen atoms of CO_2_ then coordinates at the pendant iron, which is associated with the loss of a water molecule from the iron center (***B***). The adduct is stabilized by a hydrogen bond with a protonated lysine moiety. Cleavage of the C-O single bond (***C***) results in a Ni^II^CO species, which loses CO (***D***) to regenerate [Ni] CODH and complete the catalytic cycle.

**Figure 1:**
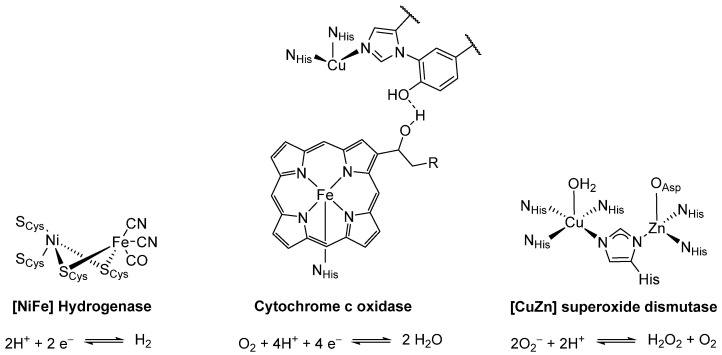
Selected multimetallic enzymes with the respective reaction they catalyze [25,26,27,28,29,30,31,32,33].

It is apparent from the catalytic cycle presented in Scheme 2 that the interaction between the two metals, Ni and Fe, is detrimental to the overall activity of the enzyme. The pendant iron is in relatively close spatial proximity (2.7 Å) to the coordinatively unsaturated Ni species [22]. This motif of cooperative catalysis is not unique to [Ni] CODH but can rather be found in multiple different enzymes catalyzing reactions with small molecules such as H_2_, N_2,_ O_2,_ or CO [7]. A few notable examples of such multimetallic metalloenzymes, including the reaction each enzyme catalyzes, are shown in Figure 1 [23,24].

While the intermetallic distance at all four active sites is relatively short, the structural motif of how these multimetallic enzymes are assembled is conceptually different. Where [Ni] carbon monoxide dehydrogenase (d_Ni···Fe_ = 2.7 Å) is a multimetallic cluster, [NiFe] hydrogenase (d_Ni···Fe_ = 2.9 Å) consists of a heterobimetallic dimer. In [CuZn] superoxide dismutase (d_Cu···Zn_ = 6.3 Å), the heterobimetallic structure is held together by a bridging imidazolato ligand, and in cytochrome c oxidase (d_Fe···Cu_ > 4.6 Å) the interaction occurs entirely through space [22,28,29,33].

Independently, with the growing understanding of intermetallic interactions and their role in enzyme catalysis, conceptually similar activation modes have been utilized in heterogeneous catalysis, usually referred to as promoter-modified metallic surfaces. In these catalytic systems, small molecules are activated by multiple metal centers through cooperative mechanisms, thus enabling processes such as steam reforming, ammonia synthesis, and catalytic converters [34,35,36,37].

**Figure 2:**
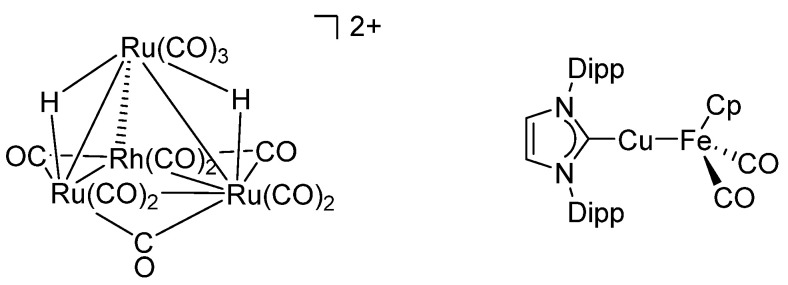
(**Left**) [H_2_Ru_3_Rh(CO)_12_]^2+^ as an example of an early heterobimetallic cluster [45]. (**Right**) [CuFe] heterobimetallic complex, utilizing σ-donating and π-accepting ligands [54].

However, in synthetic homogeneous catalysis, the main paradigm was the development of single-site catalysts. To avoid the formation of multimetallic assemblies, elaborate ligand scaffolds were designed, synthesized, and used with great success [38]. Forming bi- or polynuclear complexes was even described as a possible deactivation pathway [39,40]. The field of homogeneous multimetallic catalysis has its origins in the works of Muetterties and his concept of cluster surface analogy [41,42,43]. Here, the properties of metallic surfaces were mimicked by low-valent, often late-transition metal cluster compounds, many of which used simple ligands such as carbonyl, hydride, or phosphine ligands (Figure 2, left) [43,44,45,46,47]. More recently, σ-donating and π-accepting ligands have been employed to form various heterometallic complexes for small molecule activation [48,49,50,51,52,53]. One example is Mankad’s bimetallic catalytically active complex [(^Dipp^NHC)Cu-FeCp(CO)_2_] (^Dipp^NHC = *N,N′*-bis(2,6-diisopropylphenyl)imidazole-2-ylidene, Figure 2, right) [54]. However, all these complexes use ligand systems developed for, or commonly employed in, mononuclear transition metal chemistry. Motivated by the great success of targeted ligand design, specific scaffolds have been developed to not only control the stereoelectronic environment of the metal centers but also allow specific interactions between them and thus enable new reactivities or activation modes of small molecules [55,56,57,58,59,60].

Considering that the distinction between a cluster, a multimetallic assembly, and a polymetallic complex can be difficult and that the combinatorial possibilities between metals are virtually endless, the focus of this article is on heterobimetallic complexes with well-defined ligand scaffolds.

## 2. Heterobimetallic Complexes in Homogeneous Catalysis

Introducing a second metal into a well-defined transition metal complex has two main advantages from a catalytic point of view. First, it allows for an additional parameter with which to influence the overall reactivity of the system. Second, it allows new reaction pathways that are inaccessible to single-site catalysts [58]. In the following text, these unique reaction pathways and catalytic mechanisms will be presented. The focus will be on the catalytic application of heterobimetallic complexes and the opportunities they offer. Stoichiometric reactions, preparatory aspects, and structural features of heterobimetallic complexes will not be the main aspects. Furthermore, as this is an underdeveloped field with a non-standardized nomenclature, both for the complexes themselves and the types of mechanisms, this article, while thorough, is not comprehensive.

Heterobimetallic catalysts can be classified according to their interaction with the substrate or substrates. This mode of classification was first proposed by Page, Walker, and Messerle and later picked up and specified by Mankad and Tomson et al. [59,61]. In the following, a slight variation of these classification systems will be proposed and used throughout this work [55].

**Figure 3:**
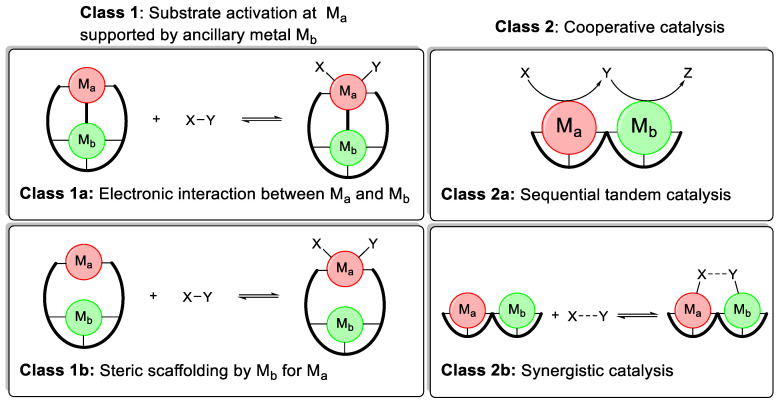
Classification of heterobimetallic catalysts by interaction with substrates.

In general, there are two classes of heterobimetallic catalysts (Figure 3). Class 1 systems have one metal M_a_, performing substrate activations and transformations, with the second metal M_b_ playing an auxiliary role. Within class 1 systems, two types of auxiliary interactions are possible [61].

***Class 1a (Figure 3, top left):*** All transformations of the substrate(s) are performed by M_a_. The second metal M_b_ has an electronic interaction with the active metal M_a_. This stereoelectronic influence could either be via a direct metal-metal bond or through a shared ligand group. This is the most commonly employed strategy in heterobimetallic catalysis [53,62,63].

***Class 1b (Figure 3, bottom left):*** All transformations of the substrate(s) are performed by M_a_. The second metal M_b_ directs the substrates into an advantageous alignment but does not participate directly in the bond formation/breaking process [64]. This strategy is most common in asymmetric heterobimetallic catalysis, significantly increasing the selectivity [60,65].

In class 2 systems (Figure 3) on the other hand, both metals M_a_ and M_b_ interact with the substrate(s) and participate in the bond-breaking and bond-formation process. Depending on when and how they participate in the reaction, they can be subdivided into two further categories.

***Class 2a (Figure 3, top right):*** Each metal performs a separate transformation on the substrate, usually in a subsequential or tandem fashion. M_a_ can transform substrate X into Y, which is then the substrate for a different reaction to Z catalyzed by M_b_. This type of cooperative interaction has received increasing attention in the past few years [57,66].

***Class 2b (Figure 3, bottom right):*** Both metals M_a_ and M_b_ participate in the bond-breaking and bond-formation processes of one single reaction. This could be achieved through the simultaneous activation of one substrate by both metals, thus lowering the activation barrier of one elemental step even further. Alternatively, substrate X is activated by M_a_ while substrate Y is activated by M_b_, resulting in a double activation process. Both will be referred to as *synergistic catalysis or activation* throughout this work. A synergistic mechanism is the least utilized of the four possible heterobimetallic mechanisms [67,68].

All these interactions depend on the spatial proximity of the two metals. An intermetallic distance of 3.5 to 6 Å is considered ideal [69]. Heterobimetallic mechanisms are also not mutually exclusive. Particularly, electronic interactions are very common even in systems where the overall reactivity is dictated by tandem catalysis. Due to a very short intermetallic distance and/or shared conjugated ligand systems, M_b_ can influence the stereoelectronic properties of M_a_, increasing its activity. It is further necessary to point out that there are many heterobimetallic catalysts for which the mechanisms are not well understood, or it is not even clear if the active species is truly a heterobimetallic complex, further complicating classification. In the following sections, each of these four classes will be elucidated with a few selected examples of heterobimetallic catalysts for which the mechanism has been expounded. While most of these systems are well studied, the homogeneity of the reaction has not been assessed for every single example presented. However, considering the in-depth mechanistic investigation conducted for almost all of the presented examples, the formation of cluster compounds or nanoparticles should have been detected.

### 2.1. Electronic Interaction between M_a_ and M_b_

In 1964, Cotton described the first metal-metal multiple bond, sparking vibrant and active research in the field [70]. Inspired by the possibilities that metal-metal bonds offer in terms of new reactivities, a plethora of compounds has been synthesized and studied [71,72]. Of particular interest in this work are catalytically active heterobimetallic compounds.

**Scheme 3:**
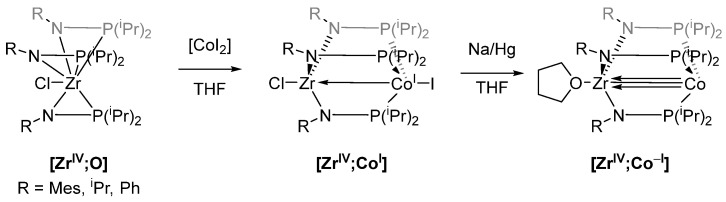
Synthesis of complex **[Zr^IV^;Co^I^]**; THF = tetrahydrofuran.

**Scheme 4:**
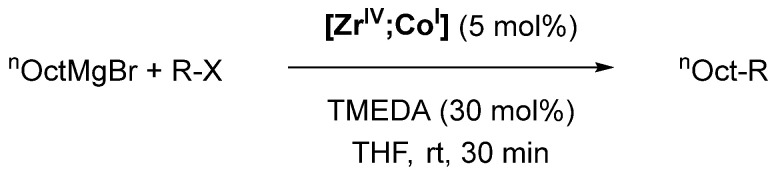
Kumada cross-coupling catalyzed by **[Zr^IV^;Co^I^]**; TMEDA = *N,N,N′,N′*-tetramethylethylendiamine [74].

A prominent example of novel reactivities and reaction mechanisms enabled by heterobimetallic complexes was reported by Thomas et al. [73,74]. Complex **[Zr^IV^;Co^I^]** (**[M_a_;M_b_]** refers to a heterobimetallic complex in which M_a_ is located in one pocket of the ligand and M_b_ in the other pocket) is obtained by reacting CoI_2_ with metalloligand **[Zr^IV^;O]** (Scheme 3), which was already employed for the synthesis of similar heterobimetallic **[Zr^IV^;Cu^I^]** and **[Zr^IV^;Mo^0^]** systems by Nagashima [75]. Interestingly, the Co center undergoes in-situ reduction from Co^II^ to Co^I^ in complex **[Zr^IV^;Co^I^]** [73]. This reduction does not occur when CoI_2_ is reacted directly with three equivalents of the phosphinoamine Ph_2_PNH(^i^Pr), indicating a reduction-aiding behavior of the Zr^IV^ center in this reaction. It is proposed that one iodide ion acts as a reductant, assisted by the Lewis acidic Zr^IV^ center in proximity to the Co ion. The resulting **[Zr^IV^;Co^I^]** complexes have Zr-Co distances from 2.628 to 2.731 Å confirmed by X-ray crystallography, showing a Co→Zr interaction. The presence of an interaction was also supported by cyclic voltammetry, where all three compounds (shown in Scheme 3) had comparatively low reduction potentials (−1.65 V to −2.05 V vs. FcH/FcH^+^) due to the electron density withdrawn from Co by Zr [73]. More interesting than the unusual metal-metal bond is the catalytic activity of **[Zr^IV^;Co^I^]** towards Kumada cross-coupling reactions (Scheme 4) [74].

Palladium-catalyzed cross-coupling reactions of aryl and vinyl halides are ubiquitous methods in preparative organic chemistry, but cross-coupling reactions between two alkyl compounds remain challenging up to this day [76]. Particularly Co-based complexes have emerged as promising catalysts for Kumada cross-coupling reactions between Grignard reagents (R′MgCl or R’MgBr; R′ = alkyl, aryl) and alkyl halides, but are not able to employ more readily available alkyl chlorides [77,78,79].

**Scheme 5:**
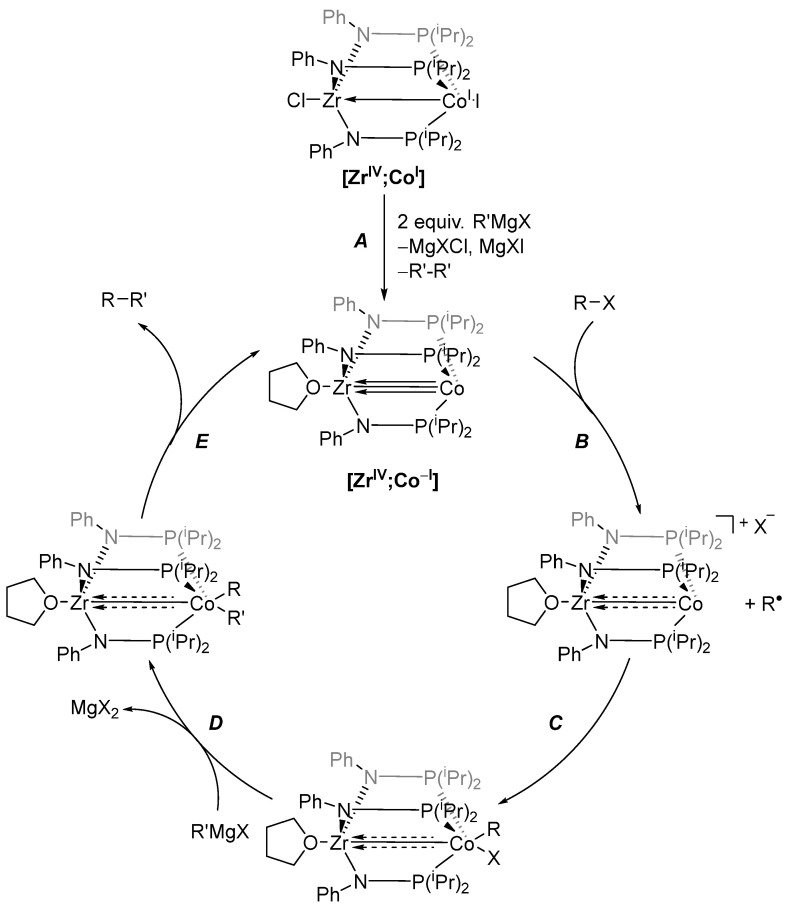
Proposed coupling mechanism catalyzed by heterobimetallic Zr/Co complexes. Dashed arrows indicate an uncertain bonding situation in the corresponding complex, as it was not isolated and thus not characterized [74].

As for the mechanism of the reaction, the precatalyst **[Zr^IV^;Co^I^]** is first reduced to the catalytically active species **[Zr^IV^;Co^−I^]** via a reaction with two equivalents of R’MgX (Scheme 5, ***A***). The active catalyst can also be obtained by direct reduction of **[Zr^IV^;Co^I^]** with sodium amalgam (Scheme 3). X-ray crystallographic characterization of the active catalyst **[Zr^IV^;Co^−I^]** revealed a very short Co-Zr distance of 2.4112(3) Å and thus an even stronger interaction between the two metals compared with **[Zr^IV^;Co^I^]**. Furthermore, density functional theory (DFT) calculations showed that this interaction occurs through σ- and π-orbital overlaps [73,80].

After the formation of the active catalyst, the addition of the alkyl halide proceeds via single electron transfer (SET, ***B***) to the alkyl halide, yielding a radical that rapidly recombines with the oxidized complex (***C***). Using (2,2,6,6-tetramethylpiperidin-1-yl)oxyl (TEMPO) as a radical trap, the formation of alkyl radicals in the mixture was confirmed. After this formal oxidative addition, a transmetallation (***D***) with the Grignard reagent R’MgX takes place. It is followed by a reductive elimination (***E***), regenerating the active catalyst and liberating the desired product R-R’. During the reductive elimination, the Zr plays a pivotal role by withdrawing electron density and aiding the rate-determining step (RDS) of the reaction [74]. This pivotal role is further proven by replacing Zr with its heavier homologue Hf. Analogous **[Hf^IV^;Co^I^]** showed significantly diminished activity, most likely due to weaker electron withdrawal from Co by Hf compared to Zr. This was confirmed through an elongation of the Co-Hf bond of 0.04 Å compared with the Zr-Co bond [81].

**Scheme 6:**
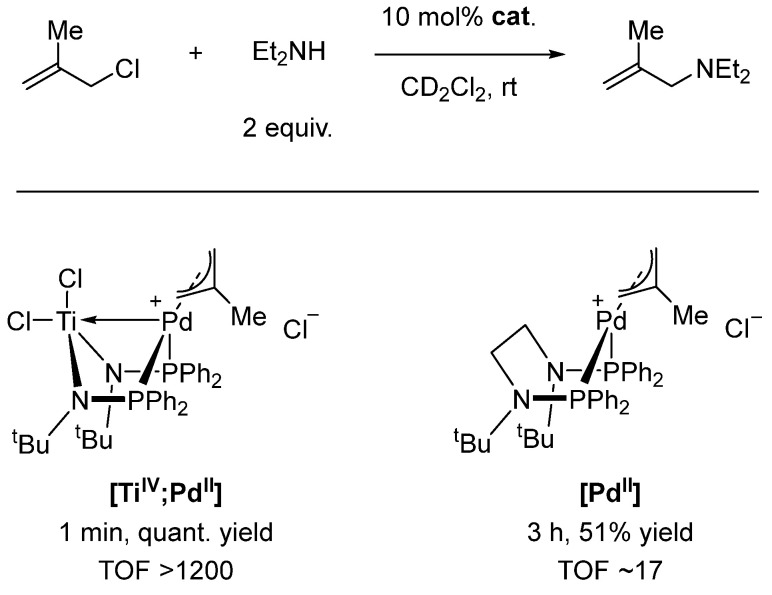
Allylic amination catalyzed by heterobimetallic **[Ti^IV^;Pd^II^]** or monometallic **[Pd^II^]** complex [83].

Employing the same ligand system and an overall similar structural motif in **[Ti^IV^;Pd^II^]**, Nagashima showed the high catalytic activity of this complex for allylic amination reactions (Scheme 6) [82]. Ess and Michaelis followed up on these findings, utilizing computational studies to provide a plausible mechanism. They could also show experimentally that the structurally similar monometallic **[Pd^II^]** is significantly less active by comparing the Turnover Frequency (TOF), which is 70 times higher for **[Ti^IV^;Pd^II^]** compared with **[Pd^II^]** (Scheme 6) [83]. Michaelis further optimized the reaction conditions and broadened the substrate scope to sterically hindered secondary amines, further underpinning the advantage of heterobimetallic **[Ti^IV^;Pd^II^]** compared with monometallic **[Pd^II^],** which can only utilize sterically unencumbered amines [84].

**Scheme 7:**
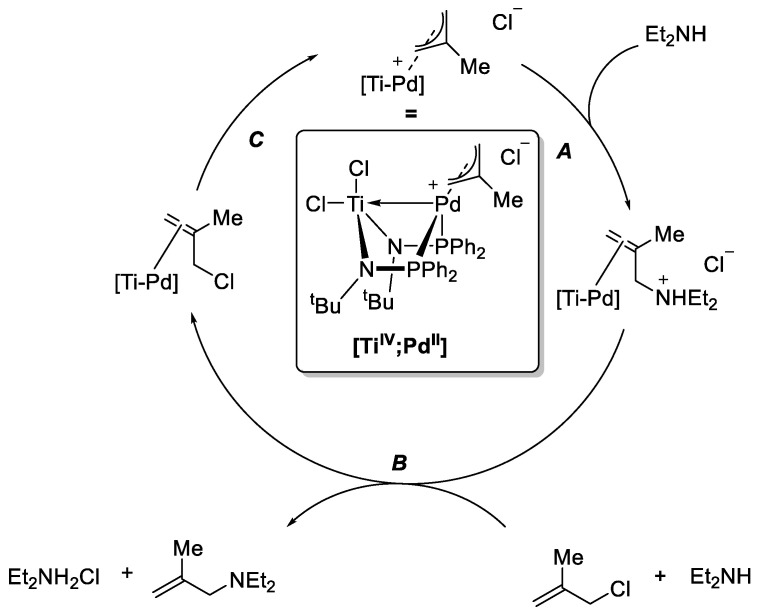
Calculated mechanism for the allylic amination catalyzed by heterobimetallic **[Ti^IV^;Pd^II^]** [82,83].

Mechanistically, the secondary amine adds nucleophilically to a terminal *η*^3^-allyl carbon atom (Scheme 7, ***A***). This results in the reduction of Pd^II^ to Pd^0^. The allyl ammonium intermediate dissociates and is deprotonated by a second amine equivalent, while an allyl chloride coordinates at the Pd center (***B***). A backside attack of the Pd^0^ at the carbon-chloride bond regenerates the catalyst (***C***). The RDS of the reaction is the addition of the amine to the catalyst. Similar to **[Zr^IV^;Co^−I^]**, the Lewis acidic Ti^IV^ withdraws electron density from the electron-rich transition state, thus lowering it by ~33 kJ mol^−1^, compared with **[Pd^II^]** (Scheme 6). X-ray structural analysis also gave experimental proof of this metal-metal interaction [83].

Interestingly, calculations indicate that this heterobimetallic interaction is stronger when both metals are from the same row of the periodic table, meaning that a Ni analogue of **[Ti^IV^;Pd^II^]** should show even higher catalytic activity than its Pd counterpart [85]. While the catalytic activity of such Ni systems was conceptually proven, no optimization of catalytic conditions was completed [82].

**Figure 4:**
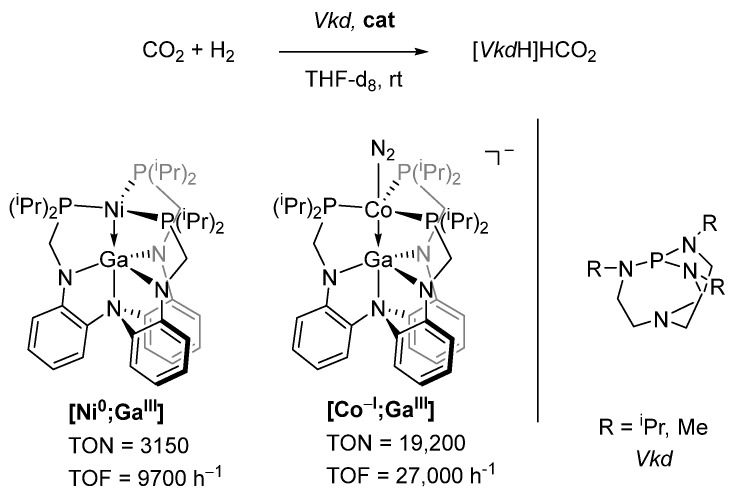
Carbon dioxide hydrogenation to formate salts, catalyzed by heterobimetallic complexes **[Ni^0^;Ga^III^]** and **[Co^−I^;Ga^III^]** [86,87,88].

Lu et al. utilized a double pincer-type ligand to develop a whole class of complexes, among them heterobimetallic **[Ni^0^;Ga^III^]** and **[Co^−I^;Ga^III^]** (Figure 4) [86,87,88]. Both of these complexes were found to be active catalysts for homogeneous carbon dioxide hydrogenations to formate salts when a super basic proazaphosphatrane base (*Vkd*, Verkade base, Figure 4) was employed. While **[Ni^0^;Ga^III^]** achieved impressive turnover numbers (TON) and turnover frequencies (TOF) for a Ni-based catalyst, it was outperformed by **[Co^−I^;Ga^III^]**. 

**Scheme 8:**
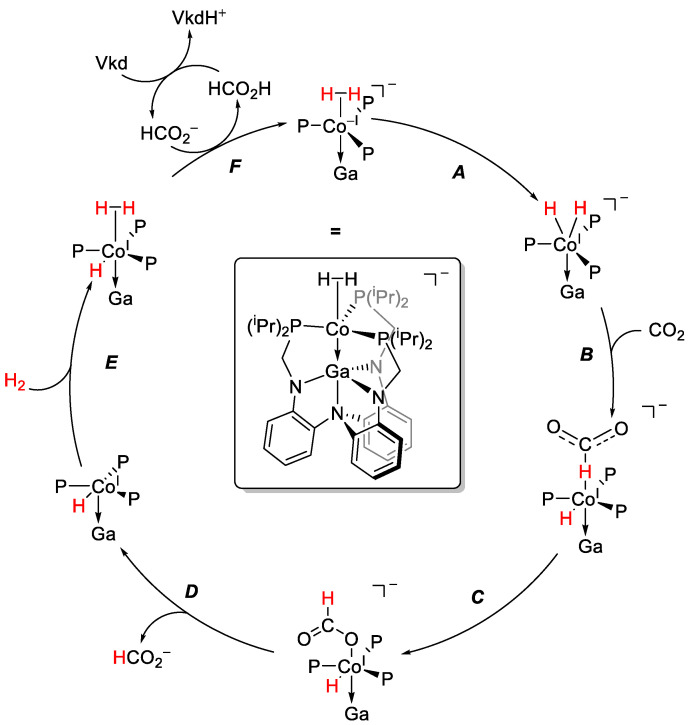
Proposed catalytic cycle for carbon dioxide hydrogenation, using **[Co^−I^;Ga^III^]** as catalyst and *Vkd* as a base [87].

By isolating reactive intermediates and performing in-situ high-pressure NMR (HP-NMR) spectroscopic and theoretical studies, the mechanism presented in Scheme 8 was proposed. The dinitrogen ligand in **[Co^−I^;Ga^III^]** is readily replaced by hydrogen, forming a masked dihydride in which the oxidative addition of dihydrogen to Co^−I^ occurs readily (***A***). This is followed by the RDS, the hydride transfer to CO_2_ (***B***), and quick isomerization to the O-bound formate complex (***C***). After the liberation of the formate anion (***D***), dihydrogen binds to the Co^I^ intermediate (***E***). A deprotonation of the complex by a formate anion (***F***) formally reduces the Co^I^ center back to a Co^−I^_,_ thus closing the catalytic cycle. Interestingly, the non-ionic superbase *Vkd* does not participate directly in the mechanism due to steric interactions but rather participates in a secondary cycle, deprotonating formic acid and thus generating a smaller base in situ that reacts readily with the complex.

Replacing Ga^III^ with Al^III^ or In^III^ lowered the activity significantly. While an aluminum analogue still had diminished activity, the indium complex was inactive. Comparing the reactivity of the corresponding intermediates showed that for the **[Co^−I^;Al^III^]** complex, the hydride transfer to carbon dioxide is thermodynamically less favorable, as is deprotonation for regeneration of the active catalyst. This emphasizes the significance of the *Z*-type interaction between the two metals [87].

### 2.2. Steric Scaffolding by M_b_ for M_a_

**Figure 5:**
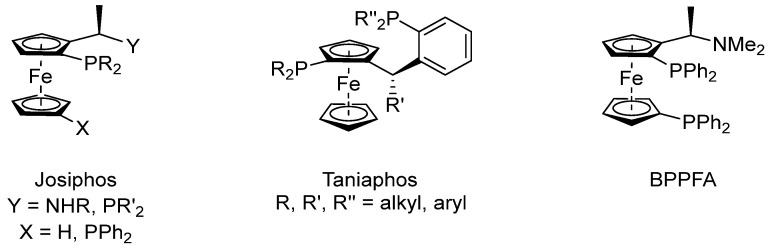
Structures of Josiphos, Taniaphos and BPPFA [94,95,96].

The concept of a metalloligand is quite common in preparatory organic chemistry. Ferrocene-based ligand systems are, for example, employed in asymmetric allyl substitution reactions together with copper or palladium pre-catalysts, asymmetric aldol reactions catalyzed by gold, and asymmetric hydrogenations, to name just a few [89,90,91,92]. Ferrocene as a ligand scaffold holds many advantages, such as high stability, both chemically and thermally, a rigid structure, ease of substitution, and planar chirality. Particularly, the latter is ideal for asymmetric catalysis, forcing substrates into a certain alignment and thus achieving asymmetric induction. Their recognition led them to industrial applications of up to 10,000 tons a^−1^ [93]. Notable examples of ferrocene-based ligands are Josiphos, Taniaphos, and BPPFA (Figure 5). However, in most reactions, the active catalyst is not isolated or even identified [94,95,96].

Contrary to ferrocene-based systems, there are well-defined catalyst classes that use M_b_ for steric scaffolding, according to Figure 3. While electronic interactions (class 1a, Figure 3) are dominated by *Z*-type interactions between the metalloligand M_b_ and the active metal M_a_, steric scaffolding is dominated by a Lewis acidic interaction between a substrate and M_b_ aligning it for the reaction [60,65].

**Scheme 9:**
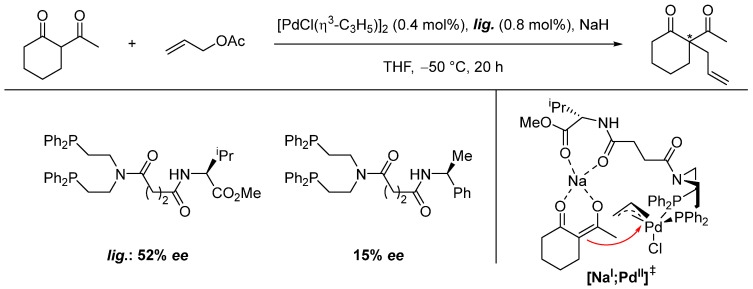
Enantioselective allylic alkylation and proposed mechanism with transition state **[Na^I^;Pd^II^]^‡^** for asymmetric induction (* refers to a stereogenic center) [60,97].

The first pioneering efforts to use heterobimetallic complexes for asymmetric catalysis were completed by Kumada, who developed a chiral ligand for asymmetric palladium-catalyzed allylic alkylation of 1,3-diketones (Scheme 9) [97]. Using an additional chelating function significantly improved the enantioselectivity (52% *ee* vs. 15% *ee*), as it puts the alkali metal ion in a crucial position to enhance the stereoselectivity. Although the active catalyst was never isolated and no in-depth mechanistic studies were undertaken, Park and Hong rationalized the asymmetric induction via a heterobimetallic transition state **[Na^I^;Pd^II^]**^‡^ [60].

**Scheme 10:**
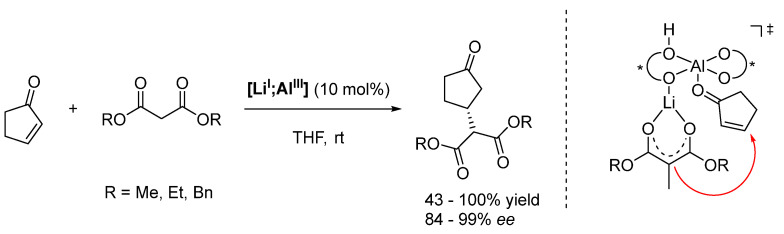
Asymmetric Michael addition catalyzed by heterobimetallic complex **[Li^I^;Al^III^]** (* refers to a stereogenic element) [109,111,112].

**Figure 6:**
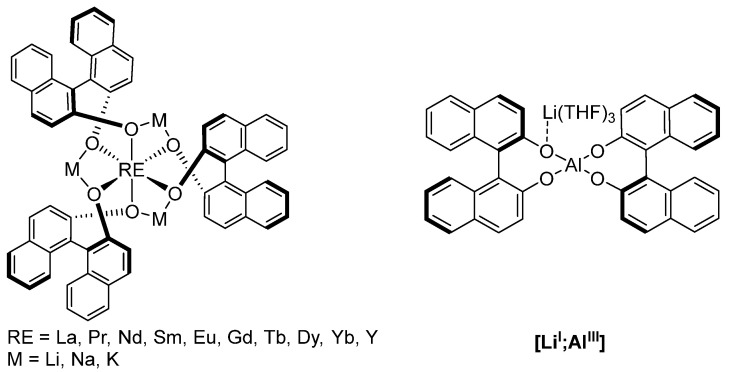
BINOL-based multimetallic catalysts (**left**) and heterobimetallic **[Li^I^;Al^III^]** catalyst (**right**) [98,99,100,101].

Following these explorative studies, Shibasaki and coworkers developed a whole class of 1,1′-bi-2-naphthol (BINOL)-based complexes (Figure 6) and demonstrated their versatility [98,99,100,101]. Particularly the multimetallic rare earth (RE)-alkali metal complexes (Figure 6, left) can catalyze a variety of reactions, such as nitroaldol reactions, conjugate addition of malonates, aza-Henry reactions, and direct aldol reactions [102,103,104,105,106,107]. Later, a heterobimetallic **[Li^I^;Al^III^]** complex was successfully employed for conjugate additions of malonates (Scheme 10) and 1,4-addition of Horner-Wadsworth-Emmons reagents [108,109,110].

The mechanism is exemplified by the conjugate additions of malonates presented in Scheme 10. The Brønsted basic ligand backbone deprotonates the malonate and thus generates the nucleophile; the Lewis acidic Al center activates the electrophile. Meanwhile, the stereoselectivity is generated by the Li ion, which directs the malonate into an advantageous position [111,112].

### 2.3. Sequential Tandem Catalysis

A fundamentally different approach to heterobimetallic catalysis, from a conceptual point of view, is to utilize the reactivity of both metals instead of using M_b_ as an extension of the ligand system for M_a_. As presented in Figure 3, there are two different approaches to involving both metals directly in the bond-breaking and/or bond-formation processes. One is via tandem catalysis, for which heterobimetallic complexes are particularly suitable as they can incorporate two different metal centers that can each perform a different type of reaction. Tandem reactions are especially interesting as they can reduce the amount of contamination, purification, and solvent involved in a multistep process [113,114,115,116].

Hahn and Peris have made major contributions in this field. Utilizing 1,2,4-triazol-di-ylidene (*ditz*) as a di-carbene ligand, they obtained a series of heterobimetallic complexes consisting of two different platinum group metals, studied their catalytic behavior in various reactions, and analyzed the interaction between the metals [57,66]. A few notable examples will be given here, demonstrating the versatility of their approach.

**Scheme 11:**
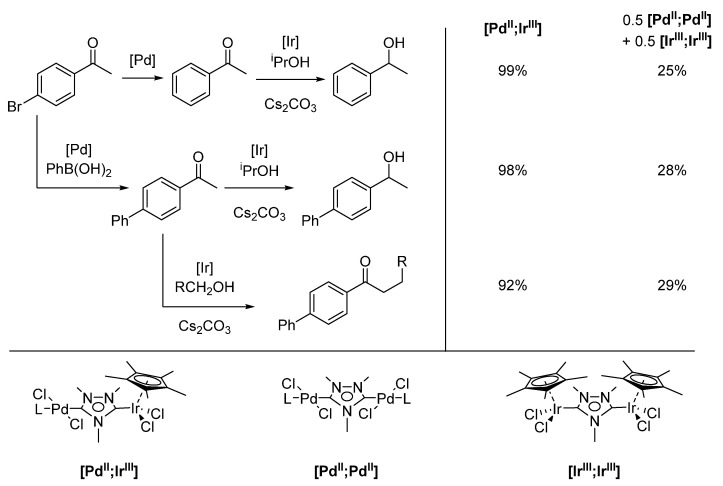
Heterobimetallic complex **[Pd^II^;Ir^III^]** and its homobimetallic counterparts, **[Pd^II^;Pd^II^]** and **[Ir^III^;Ir^III^]**, (L = pyridine, MeCN) and the respective yields for three tandem reactions: Dehalogenation/transfer hydrogenation reactions (**top**), Suzuki-Miyaura cross-coupling/transfer hydrogenation (**middle**), and Suzuki-Miyaura cross-coupling/α-alkylation reaction (**bottom**, R = Ph, ^i^Pr, ^n^Pr) [117].

Using a **[Pd^II^;Ir^III^]** catalyst, they could perform a dehalogenation/transfer hydrogenation tandem reaction, yielding 1-phenylethanol (Scheme 11). Through variation of the reaction conditions, more complex tandem processes were possible, namely a Suzuki-Miyaura coupling/transfer hydrogenation, by introducing phenylboronic acid and a Suzuki-Miyaura coupling/α-alkylation by substituting the secondary for a primary alcohol. These reactions not only proceeded with excellent yields but also demonstrated a degree of intramolecular cooperativity between the two metals [117].

A first indication of the cooperativity between the two different metals is that a mixture of the two homobimetallic complexes performs worse than the heterobimetallic complex under the same reaction conditions. Considering that the distance between Pd and Ir in **[Pd^II^;Ir^III^]** is 6.039 Å and therefore at the upper limit of what is ideal for interaction, this is somewhat surprising [117]. Cyclic voltammetry and DFT studies of similar *ditz*-based heterobimetallic systems indicate that a certain degree of electronic interaction might be responsible. Diruthenium *ditz* complexes showed a separation of 120 mV between the oxidation peaks, which corresponds to a class II system according to the Robin and Day classification [118,119]. Using DFT, the Tolman Electronic Parameters (TEPs) of the free *ditz* ligand and *ditz* systems, in which one coordination site was occupied by a metal, were calculated [120,121]. A clear shift of the TEP value based on the second metal could be observed, further indicating an electronic interaction between the two metals [122]. However, the electronic interaction is too small to account for the strong cooperative effect in the heterobimetallic complexes, and further investigation into the nature of the interaction and the overall mechanism is still ongoing [57].

**Scheme 12:**
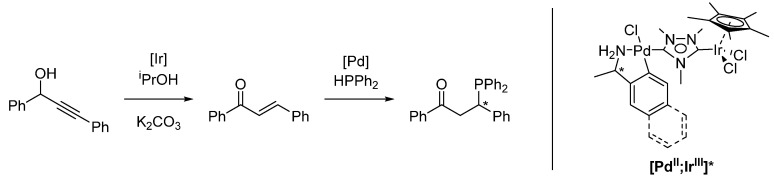
Enantioselective isomerization/hydrophosphination reaction, catalyzed by chiral [Pd^II^;Ir^III^]* (* refers to a stereogenic center) [123].

Using a chiral coligand, resulting in a chiral heterobimetallic [Pd^II^;Ir^III^]* complex, an enantioselective isomerization/hydrophosphination tandem reaction was catalyzed (Scheme 12). While the activity of the system is high and the regioselectivity is very good, the enantioselectivity is poor (max. 17% *ee*). However, to this day, this is the only example of a chiral tandem reaction catalyzed by a heterobimetallic complex [123].

**Scheme 13:**
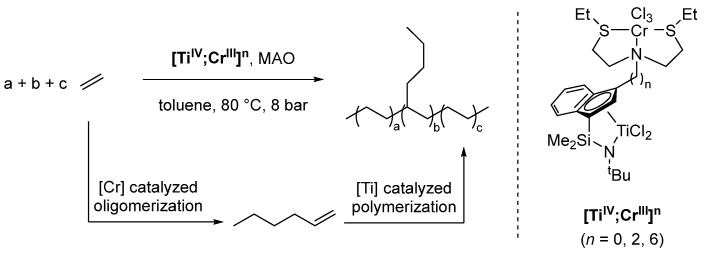
Oligomerization/polymerization tandem reaction, catalyzed by **[Ti^IV^;Cr^III^]^n^** (*n* = 0, 2, 6). *n* = 0: highest activity, highest M_n_, highest α-olefin incorporation; *n* = 6: lowest activity, lowest M_n_, lowest α-olefin incorporation [124].

Utilizing a ditopic ligand system with two very different coordination environments, Marks obtained a series of heterobimetallic **[Ti^IV^;Cr^III^]** complexes with varying distances between the two metal centers, depending on the length (*n*; *n* = 0, 2, 6) of the alkyl spacer (**[Ti^IV^;Cr^III^]^n^** (Scheme 13) [124]. These systems are active catalysts for oligomerization/polymerization tandem reactions of ethylene, resulting in linear low-density polyethylene (LLDPE) with exclusive *n*-butyl branches when methyl aluminum oxide (MAO) is employed as a cocatalyst for activating the Ti center. Here, the Cr^III^ center catalyzes the oligomerization of ethylene to 1-hexene selectively, while Ti^IV^ catalyzes the polymerization.

**Scheme 14:**
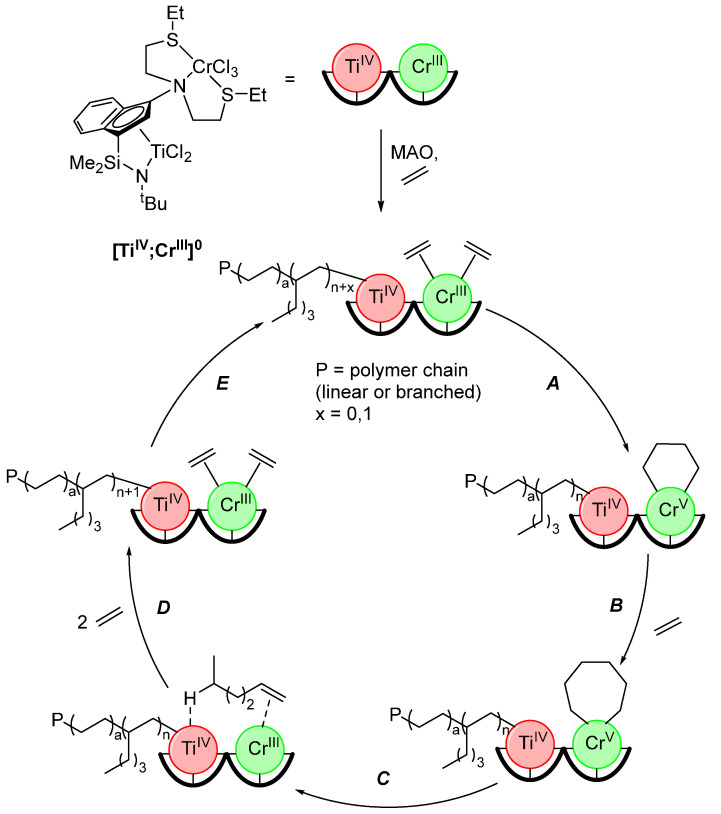
Proposed (simplified) mechanism for oligomerization/polymerization tandem reaction catalyzed by **[Ti^IV^;Cr^III^]^0^** [124].

The complex with the shortest intermetallic distance (*n* = 0) exhibited the highest activity, average molecular weight (M_n_), and branch density; all three values dropped with an increasing *n*. Compared with the mixture of the monometallic analogues, the branch density and M_n_ are higher for **[Ti^IV^;Cr^III^]^0^**. However, the overall activity is higher in the mixture of the monometallic complexes. When 1-pentene was introduced as competition for incorporation in the polymer chain against the in-situ generated 1-hexene, only 1-hexene enchainment was observed, indicating a favorable interaction between the two metals towards binding of the latter. Combined with DFT calculations, these insights lead to the proposition of the mechanism presented in Scheme 14. Multiple different pathways for the formation of 1-hexene and copolymerization with ethylene are possible, but only the main pathway is discussed. After the coordination of two ethylene molecules by the Cr^III^ center, an oxidative addition (***A***) leads to the formation of a five-membered metallacycle, which is expanded to a seven-membered metallacycle with a third ethylene molecule (***B***). Reductive elimination (***C***) leads to the formation of 1-hexene, which forms a hydrogen bridge to the Ti^IV^ center, thus positioning it ideally for incorporation into the growing polymer chain (***D***) [124].

**Figure 7:**
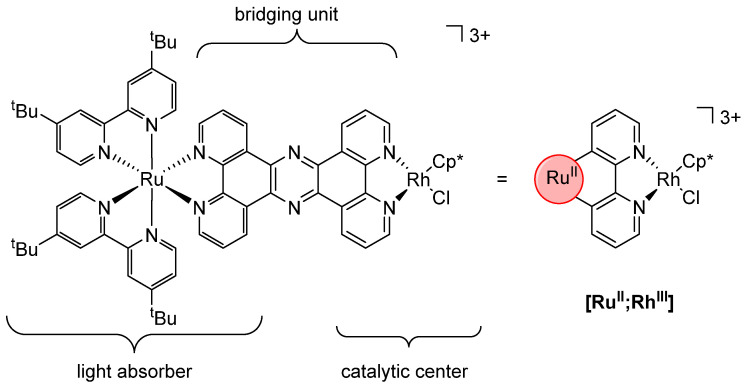
**[Ru^II^;Rh^III^]** as an example of a photochemical molecular device (Cp* = C_5_Me_5_) [129,132,133].

A different approach was taken by Rau and his coworkers. They developed photochemical molecular devices (PMDs) and used them for various reduction reactions [125,126,127,128,129,130,131,132,133]. These PMDs consist of three structural units (Figure 7): a photoactive Ru^II^ complex fragment that acts as a light absorber, a catalytic center (in the chosen example, a [Rh^III^Cp*Cl] moiety, Cp* = C_5_Me_5_), and a bridging unit that connects the two metals via a conjugated reducible π-electron system [129,133].

**Scheme 15:**
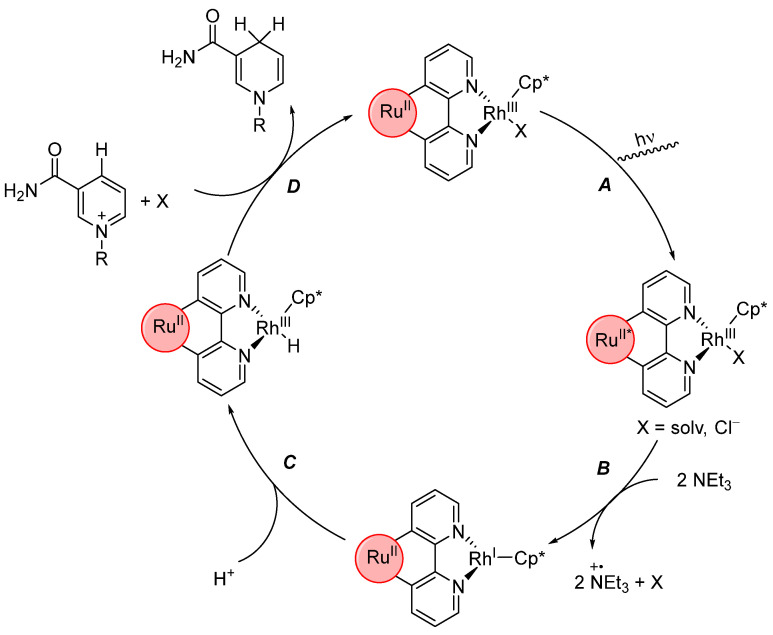
Photocatalytic reduction of nicotinamide with triethylamine as reducing agent, NaH_2_PO_4_ as proton source and **[Ru^II^;Rh^III^]** as catalyst. Corresponding charge of the complex not indicated. Oxidized triethylamine decomposes in a secondary reaction to undefined products (Cp* = C_5_Me_5_) [132].

**[Ru^II^;Rh^III^]** was used for the photochemical reduction of nicotinamide, with triethylamine as a reducing agent and NaH_2_PO_4_ as a proton source (Scheme 15) [132]. The mechanism of this reduction nicely demonstrates the underlying principle of PMDs. 

First, the Ru^II^ center is photoexcited (***A***) which enables the abstraction of an electron from triethylamine via single electron transfer (SET). The electron is then transferred to the Rh^III^ center via the reducible bridging unit. Replacing the bridging unit with a simpler bipyrimidine led to a complete loss of catalytic activity, showing that the transfer of electrons from the light absorber to the catalytic center is the important step [129,133]. The photoredox reaction between the Ru^II^ center and triethylamine has to occur twice (***B***) to yield a reduced Rh^I^ species, which can then oxidatively add a proton (***C***) to generate a rhodium(III) hydride species. A hydride transfer then reduces nicotinamide. The advantage of these systems becomes clear when they are compared with their monometallic counterparts. They show diminished to no catalytic activity, as the reaction mixture is extremely diluted and two intermolecular electron transfers would have to occur, in between which the catalytically active center has to be stable [132,134,135].

### 2.4. Synergistic Catalysis

Out of the four classes of heterobimetallic complexes, synergistic complexes are the least developed (Figure 3) [68]. While the other three classes have been recognized as viable options to design novel catalysts and thus reviewed extensively, the first example of a synergistic interaction in a heterobimetallic complex was reported in 2003 by Lau [136]. However, most of the progress in this field has occurred over the last decade [68]. The potential of synergistic interactions is apparent when considering the mechanism of CO_2_ reduction by [Ni] CODH (Scheme 2). Both metals participate directly in the activation of the substrate and thus enable the overall reactivity. Reviewing this field is compounded by the inconsistent use of the term synergism. While it is narrowly defined in this work as two metals working together in the same bond-breaking and/or bond-formation process of a single reaction, it is sometimes used to describe any increase in reactivity when a heterobimetallic complex is used or to describe any kind of interaction between the two metals without further specification [57,61,118].

**Scheme 16:**
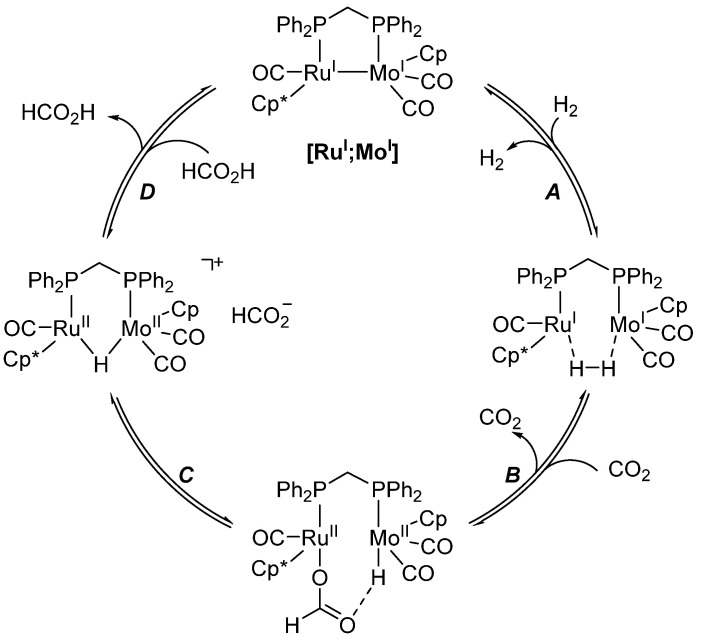
Proposed mechanism of CO_2_ hydrogenation and formic acid dehydrogenation catalyzed by **[Ru^I^;Mo^I^]** (Cp = C_5_H_5_; Cp* = C_5_Me_5_) [136].

Most synergistic heterobimetallic systems use synergistic interactions for the activation of small molecules. The very first system in which a synergistic interaction was found (**[Ru^I^;Mo^I^]**, Scheme 16) is active for both the decomposition of formic acid to carbon dioxide and dihydrogen and the reverse CO_2_ hydrogenation yielding formate salts, although both with very low TON (<43) [136].

**[Ru^I^;Mo^I^]** first activates H_2_ across the Ru-Mo bond (***A***), followed by the insertion of CO_2_ into the more hydridic Ru-H bond (***B***). The resulting complex liberates the anionic formate (***C***), which, in the last step, abstracts the bridging H atom, regenerating the catalyst (***D***). The reaction was monitored using HP-NMR, and the only species observed throughout the reaction was **[Ru^I^;Mo^I^]**. This, together with the studies completed on isolated intermediates, indicates that the low activity is due to a non-facile reaction of **[Ru^I^;Mo^I^]** with H_2_ to form the dihydride species. However, the monometallic counterparts and mixtures thereof were all inactive for the same reactions, showing that the heterobimetallic assembly is essential for catalytic activity [136].

**Scheme 17:**
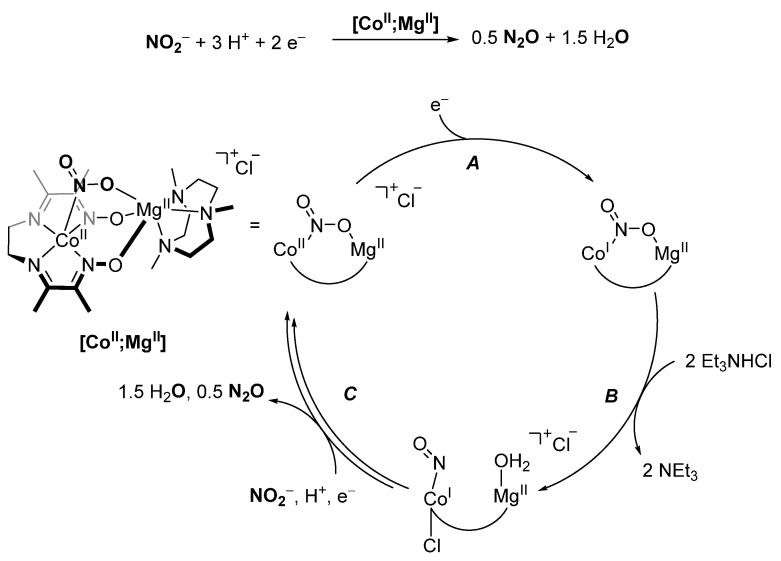
Proposed catalytic cycle for 2 e^−^/3 H^+^ reduction of NO_2_^−^, catalyzed by **[Co^II^;Mg^II^]** [137].

Inspired by heme-containing nitrite reductases, Peters and coworkers developed a heterobimetallic **[Co^II^;Mg^II^]** complex for the electrochemical reduction of NO_2_^−^ (Scheme 17). Using −1.2 V vs. SCE and Et_3_NHCl as proton sources, N_2_O was produced selectively. Based on stoichiometric reactions and isolated intermediates, a rudimentary mechanism was proposed (Scheme 17). Quintessential for the overall activity is the initial binding of nitrite by both the Mg^II^ and Co^II^ centers, as it not only activates the nitrite but also stabilizes the NO_2_^−^ adduct, which is the presumed active catalyst. A single electron reduction (***A***) activates the N-O bond, which then readily undergoes N-O bond cleavage (***B***) with a mild acid, accompanied by a significant increase in the Mg-Co distance from 3.3 Å in the reduced nitrite complex to 3.7 Å in the nitrosyl aqua complex. The nitrosyl aqua complex liberates N_2_O after successive reductions and H_2_O after further protonation (***C***) [137].

**Scheme 18:**
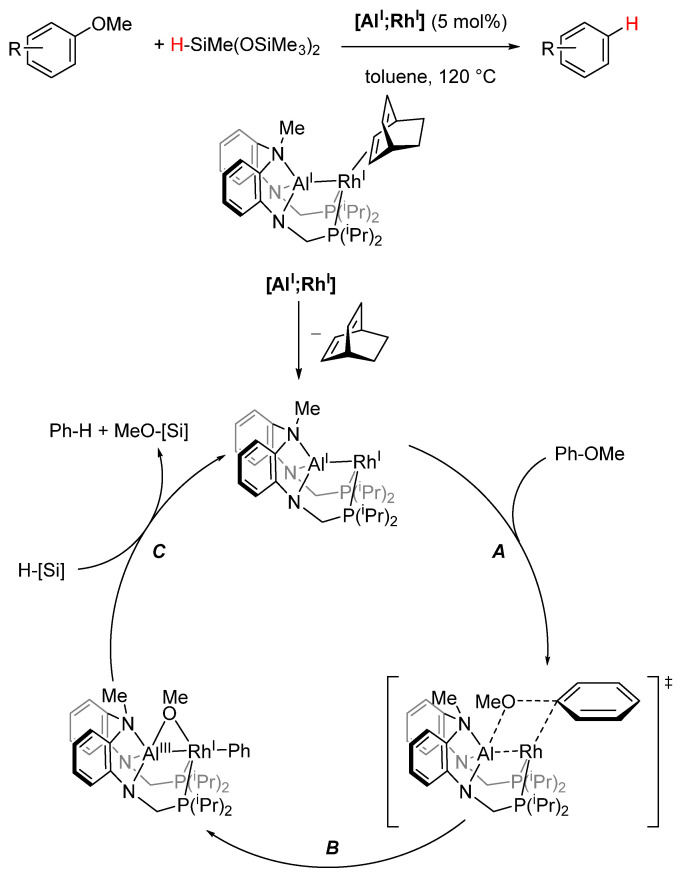
Proposed mechanism for deoxygenation reaction with silanes, using **[Al^I^;Rh^I^]** as catalyst ([Si] = SiMe(OSiMe_3_)_2_) [139].

Nakao used this principle of double activation of a single substrate to catalytically activate relatively inert C-O and C-F bonds [138,139]. Through the isolation of reactive intermediates, isotopic labeling studies, and DFT calculations, the mechanism presented in Scheme 18 was proposed. Anisole initially coordinates to the Rh^I^ center in an *η*^2^ fashion, which is directly followed by activation of the methoxy function by the Al^I^ center with simultaneous *η*^1^ coordination of the phenyl rest to the Rh^I^ center (***A***). Once the C(sp^2^)-O bond has been broken (***B***), the OMe moiety is bound as a bridging moiety, Al^III^(*µ*-OMe)Rh^I^, formally oxidizing the aluminum center. Reduction of the Al^III^ species with a silane (***C***) regenerates the catalyst and yields the final product [139].

**Scheme 19:**
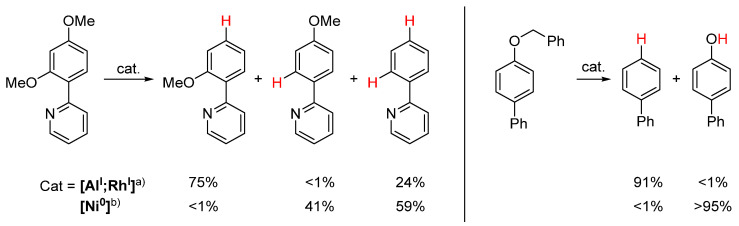
Two examples of different chemoselectivities of **[Al^I^;Rh^I^]** compared to conventional **[Ni^0^]** [139]. (a) **[Al^I^;Rh^I^]** (5 mol%), HSiMe(OSiMe_3_)_2_ (1.2 equiv.), toluene, 120 °C, 2 h. (b) [Ni(COD)_2_] (20 mol%), 1,3-bis(2,6-diisopropylphenyl)imidazolinium chloride (SIPr·HCl, 40 mol%), HSiEt_3_ (25 equiv.), NaO^t^Bu, toluene 140 °C, 3 h.

The unusual reactivity of **[Al^I^;Rh^I^]** is enabled by two types of interactions. On the one hand, the *Z*-type interaction of Al with Rh facilitates the catalytic reduction of Al^III^ to Al^I^ with mild reductants, but more importantly the simultaneous activation of the substrate at both metal centers [138]. Additionally, the sterically hindered ligand system induces orthogonal chemoselectivity compared with a conventional Ni catalyst **[Ni]^0^** ([Ni(COD)_2_] (20 mol%), 1,3-bis(2,6-diisopropylphenyl)imidazolinium chloride (SIPr·HCl, 40 mol%), Scheme 19) [139].

**Scheme 20:**
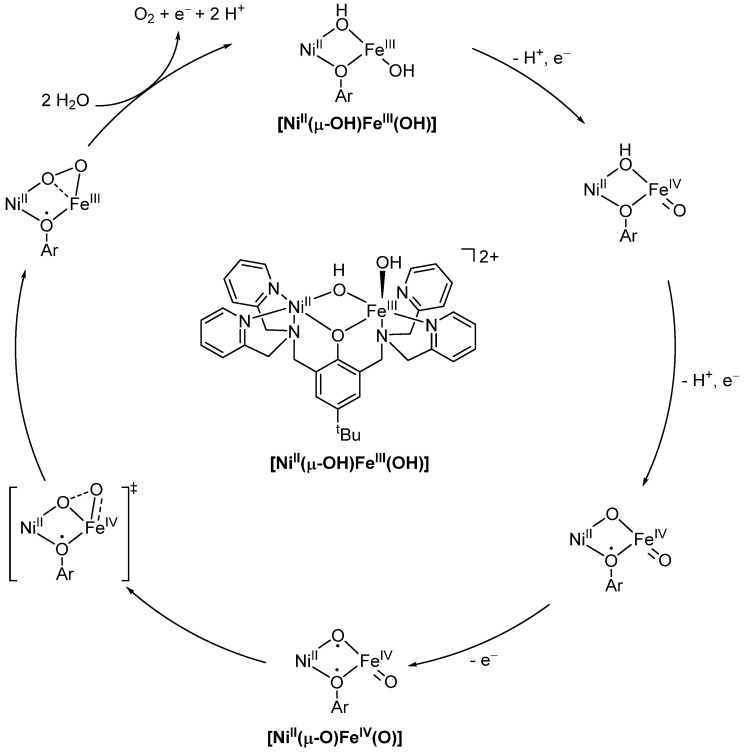
Proposed catalytic cycle for water oxidation catalyzed by **[Ni^II^(µ-OH)Fe^II^(OH)]** [140,141].

Similar to the heterobimetallic cobalt magnesium system employed by Peters et al. for nitrite reduction, Zhang and coworkers performed catalytic electrochemical water oxidation using a heterobimetallic **[Ni^II^;Fe^III^]** system (Scheme 20). While the heterobimetallic **[Ni^II^;Fe^III^]** system displayed significant catalytic activity, the monobimetallic **[Fe^III^;Fe^III^]** shows no activity, and the monobimetallic **[Ni^II^;Ni^II^]** easily breaks down into nanoparticles. Using Zn^II^ to replace either Ni^II^ or Fe^II^ and analyzing the activity of the resulting complexes indicated that the bimetallic NiFe center is responsible for the overall catalytic activity rather than one single Ni^II^ or Fe^III^ site. Using DFT calculations in combination with electrochemical measurements, the mechanism proposed in Scheme 20 was proposed. Through several single electro oxidations, the high-valent **[Ni^II^(µ-O)Fe^IV^(O)]** species is obtained. Here, the radical character of the oxygen is maintained, and thus the rate-determining oxyl-oxo coupling can occur. This pathway echoes the important role of lattice oxygen in heterogeneous NiFe catalysts [140,141].

**Scheme 21:**
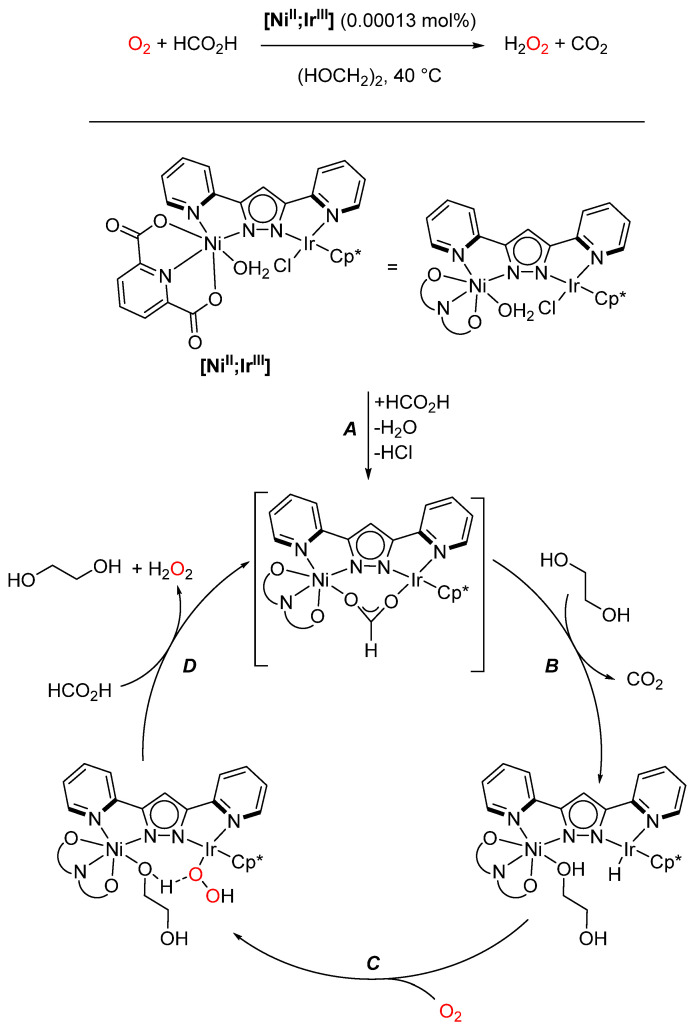
Proposed mechanism of O_2_ reduction using heterobimetallic **[Ni^II^;Ir^III^]** (Cp* = C_5_Me_5_) [142,143].

Hong and coworkers developed a series of heterobimetallic Ir^III^-based metal complexes, most notable among them the **[Ni^II^;Ir^III^]** complex depicted in Scheme 21. First, they studied their catalytic behavior towards formic acid dehydrogenation, yielding carbon dioxide and dihydrogen, and found that the heterobimetallic complexes significantly increased the rate of dihydrogen production. Of the studied complexes, the heterobimetallic **[Ni^II^;Ir^III^]** complex was the most active [142]. In the next step, the resulting hydride species was not just protonated but instead used for the reduction of oxygen. After optimizing the reaction conditions, intermediates were isolated, and kinetic analysis and DFT calculations were completed, culminating in the mechanism presented in Scheme 21. 

The active catalyst is formed by the coordination of formate (***A***) to give an Ni^II^(*µ*-O_2_CH)Ir^III^ complex, which readily releases CO_2_ (***B***) to form the hydride species on reaction with ethylene glycol. The rate-determining step is the subsequent addition of oxygen (***C***) to form the peroxyl species. Here, the Ni^II^ moiety again exerts an influence by increasing the acidity of the protic ethylene glycol coordinated at the Ni center. This facilitates the release of hydrogen peroxide coupled with the coordination of a new formate unit, regenerating the active catalyst [143]. Similar **[Cu^II^;Ir^III^]** complexes exhibited high catalytic activity for the aerobic oxidation of olefins, especially compared with their monometallic counterparts, but no indication of a synergistic mechanism was found. The increase in reactivity is probably due to a tuning of the stereoelectronic environment of the Ir^III^ center [144].

**Scheme 22:**
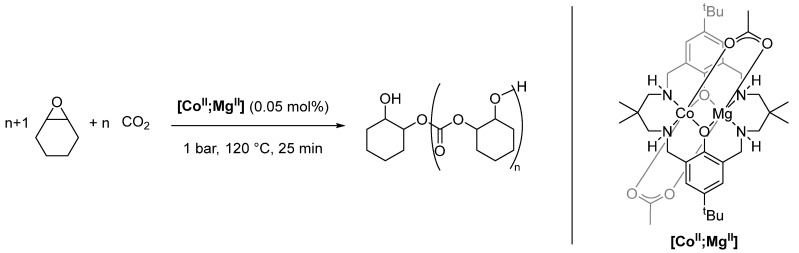
Ring-opening copolymerization of cyclohexene oxide and carbon dioxide, catalyzed by **[Co^II^;Mg^II^]** [153].

Polymerization reactions, in particular copolymerization reactions, have already utilized multimetallic assemblies [145,146]. While early works focused on homobi- and multimetallic complexes, Williams and coworkers created a group of heterobimetallic macrocyclic complexes that proved to be excellent catalysts for copolymerization reactions between epoxides and carbon dioxide [147,148,149,150,151]. Of these complexes, **[Co^II^;Mg^II^]** is the most active system and exemplifies the synergistic interaction (Scheme 22) [152,153].

**Scheme 23:**
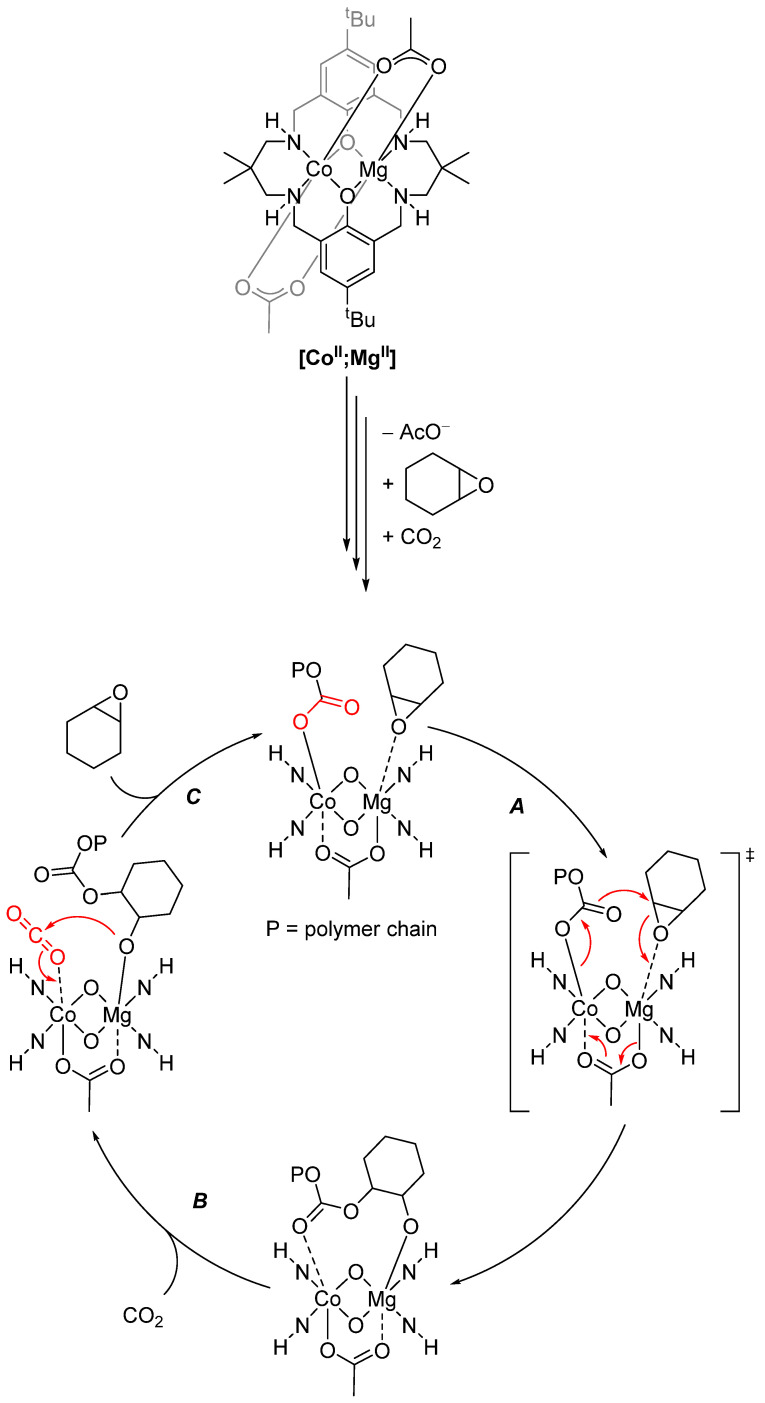
Mechanism of copolymerization of carbon dioxide and cyclohexene oxide, catalyzed by **[Co^II^;Mg^II^] [153]**.

Detailed kinetic studies showed that both metals have distinct roles in catalysis (Scheme 23). The RDS of the copolymerization is the attack of the cobalt carbonate unit on the epoxide (***A***). Here, the epoxide coordinated at the Mg center lowers the transition state entropy, favoring polymerization versus back-biting and yielding a cyclic carbonate. Meanwhile, the coordination of the carbonate at the Co center increases its nucleophilicity and thus reduces the transition state enthalpy. Carbon dioxide can replace the polymeric carbonate moiety at Co (***B***) and is then preorganized for the attack of the Mg alkoxide (***C***). This alternating chain shuttling mechanism is responsible for perfect copolymerization [153].

**Scheme 24:**
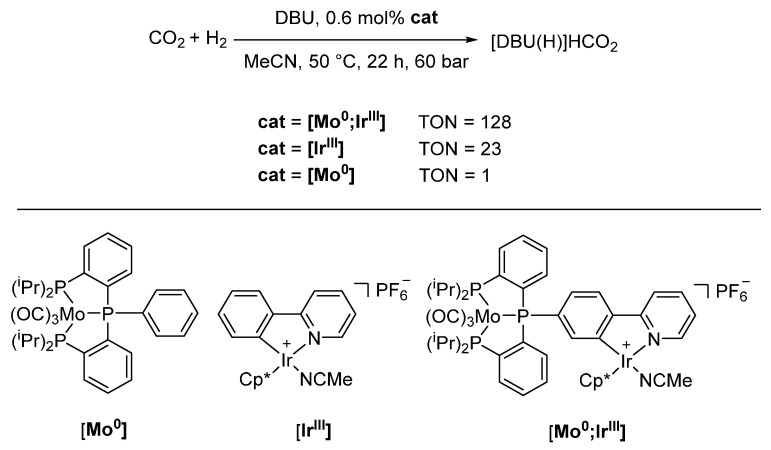
Comparison of catalytic activity of monometallic **[Mo^0^]**, **[Ir^III^]** and heterobimetallic **[Mo^0^;Ir^III^]** (DBU = 1,8-diazabicyclo[5.4.0]undec-7-ene, Cp* = C_5_Me_5_) [154].

In analogy to the first synergistic heterobimetallic system, Hey-Hawkins and coworkers published a series of heterobimetallic group 6/group 9 complexes **[M^0^;M’^III^]** (M = Cr, Mo, W; M′ = Co, Rh, Ir) and tested their catalytic activity in homogeneous carbon dioxide hydrogenation (Scheme 24). Here, **[Mo^0^;Ir^III^]** showed the highest activity and a marked increase in activity when compared with its monometallic counterparts [154,155,156].

**Scheme 25:**
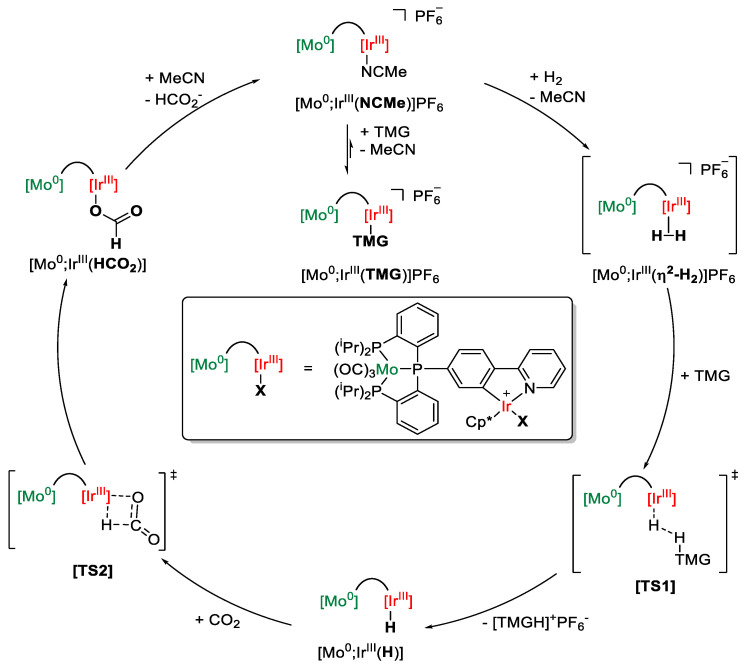
Proposed catalytic cycle for CO_2_ hydrogenation using **[Mo^0^;Ir^III^]** as catalyst and TMG as base (TMG = 1,1,3,3-tetramethylguanidine, Cp* = C_5_Me_5_) [155].

Using spectroscopic methods such as IR and ^31^P{^1^H} NMR spectroscopy, but in particular cyclic voltammetry, the two metal centers in all **[M^0^;M′^III^]** complexes were found to be electronically isolated, in contrast to all previously reported heterobimetallic complexes utilizing synergistic mechanisms [154]. The mechanism depicted in Scheme 25 was proposed based on NMR spectroscopic studies, kinetic measurements, the isolation of intermediates, and the study of their reactivity in combination with DFT calculations.

**Figure 8:**
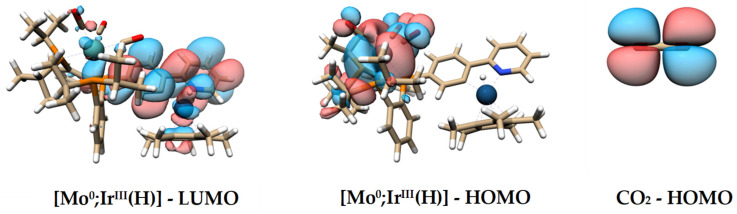
(**Left**) The LUMO of **[Mo^0^;Ir^III^(H)]**. (**Middle**) The HOMO of **[Mo^0^;Ir^III^(H)]**. (**Right**) The HOMO of CO_2_ [155].

The low activity with a TON of 128 and a TOF of only 5.0 h^−1^ can be attributed to the formation of the base adduct **[Mo^0^;Ir^III^(TMG)]PF_6,_** which lowers the concentration of the active catalyst **[Mo^0^;Ir^III^(NCMe)]PF_6_** below 1 ppm. The first productive step of the catalytic cycle is the heterolytic activation of dihydrogen via **[TS1]**, resulting in the hydride intermediate **[Mo^0^;Ir^III^(H)]**. This intermediate is the resting state of the reaction, as the following hydride transfer to CO_2_ is the rate-determining step of the reaction. The last step is the liberation of formate from **[Mo^0^;Ir^III^(HCO_2_)],** regenerating the active catalyst. DFT calculations revealed that the underlying mechanism of the reduction is a lowering of the Pauli repulsion between the HOMO of CO_2_ and the HOMO of **[Mo^0^;Ir^III^(H)]** (Figure 8). Throughout the mechanism, the octahedral Mo(CO)_3_P_3_ moiety does not participate actively in the reaction but only plays an auxiliary role in the rate-determining step by optimizing the orbital interaction between the active center and the substrate, thus increasing the reactivity [155]. 

## 3. Conclusions

The advantage of heterobimetallic catalysts over their monometallic counterparts has long been established, and, while not comprehensive, this review presents an overview of mechanistic aspects for this exceptional class of compounds. As illustrated by the examples, the increase in activity can be attributed to interactions between the two metal centers. Depending on how the metals interact with the substrates, the catalyst can be classified. While an increase in activity by using the second metal as a metalloligand or an increase in selectivity by using it to provide scaffolding are well established, using the two metals in a cooperative way is still underdeveloped. However, using heterobimetallic catalysts in a cooperative fashion enables unique reactivity and reactions and will undoubtedly be the future of this field.
